# Epilepsie et santé de la reproduction: enjeux et perspectives

**DOI:** 10.11604/pamj.2019.34.81.19366

**Published:** 2019-10-11

**Authors:** Glorien Jemissair Lemahafaka, Sonia Maminirina Fenomanana, Alain Djacoba Tehindrazanarivelo

**Affiliations:** 1 Service Neurologie, CHU Antanambao, Tuléar, Madagascar; 2 Service Gynécologie Obstétrique, CHU Antanambao, Tuléar, Madagascar; 3 Service Neurologie, CHU Befelatanana, Antananarivo, Madagascar

**Keywords:** Epilepsie, femmes, prise en charge, Epilepsy, women, treatment

## Abstract

L'épilepsie complique la vie reproductive d'une femme. La modification physiologique de la femme et les effets des antiépileptiques sont en générale la cause. Plusieurs complications peuvent s'ajouter au cours du traitement qui nécessite une surveillance rigoureuse. Les antiépileptiques entrainent surtout des effets tératogènes et malformatives. Pour éviter ou minimiser ces complications, les femmes épileptiques doivent être accompagnées dès l'âge pubertaire, en cas de la contraception associée pendant la grossesse et l'accouchement. Toujours prescrire le traitement antiépileptique en monothérapie en évitant les médicaments à effets connus tératogènes et de préférence les anciens médicaments. La prise en charge conjointe par les neurologues et gynéco-obstétriciens est plus efficaces. Pendant la grossesse, il est important de mettre la patiente sous acide folique. L'administration de vitamine K, en fin de grossesse et en période néonatale, est une prévention des complications hémorragiques périnatales. Le choix d'allaitement est individuel car il n’y a aucune contre-indication formelle de l'allaitement chez les épileptiques sous traitement. Le respect de ses conditions renforce la confiance et favorise une vie reproductive rassurante chez nos épileptiques.

## Abstract (en)

Epilepsy complicates a woman's reproductive life. The physiological modification of the woman and the effects of antiepileptic drugs are generally the cause. Several complications may be added during treatment that requires careful monitoring. Antiepileptic drugs mainly cause teratogenic and malformative effects. To minimize these complications, epileptic women should be accompanied at pubertal age, in case of the associated contraception during pregnancy and delivery. Always prescribe antiepileptic therapy as monotherapy, avoiding known teratogenic drugs and preferably old drugs. Joint care by neurologists and gynecologists and obstetricians is more effective. During pregnancy, it is important to put the patient on folic acid. The administration of vitamin K, in late pregnancy and in the neonatal period, is a prevention of perinatal hemorrhagic complications. The choice of breastfeeding is individual because there is no formal contraindication of breastfeeding in epileptics under treatment. Respecting its conditions builds confidence and promotes a reassuring reproductive life for our epileptics

## Perspective

Les épilepsies sont un ensemble de syndromes et de maladies caractérisées par une prédisposition du cerveau à présenter des crises épileptiques et par les conséquences neurobiologiques (cognitive, psychologique et sociale) qui en découlent [1]. La santé de la reproduction (OMS) c’est un état de bien-être général, tant physique que mental, social de la personne humaine pour tout ce qui concerne l’appareil génital, ses fonctions, son fonctionnement et non seulement l'absence de maladies ou d’infirmités [2]. Les résultats des différentes enquêtes permettent d'affirmer que l'épilepsie est une maladie universelle (elle peut toucher n'importe quel individu), ubiquitaire (elle existe dans tous les pays) mais inégalitaire car sa distribution selon l'âge, le sexe, la race et certains pays est différente [3]. Il est important de savoir que les patientes épileptiques devront faire face toute leur vie à des aspects spécifiques liés à la fois aux effets de la répétition de la crise et des médicaments antiépileptiques. Ces spécificités agissent sur leur fonction hormonale, leurs sexualités ou leurs désirs de reproduction. Du coté pratique, la prise en charge d’une patiente épileptique en diffère clairement de celle d’un patient épileptique. La difficulté réside tant chez les neurologues et les gynéco-obstétriciens. L'adaptation posologique et les choix des médicaments ont une importance capitale dans la prise en charge. L'objectif de cet article était de décrire les enjeux important sur la prise en charge des femmes épileptiques afin de dégager nos perspectives à venir.

**Epilepsie et puberté:** l'adolescence se situe autour de 11 à 13 ans. C'est une période de modification du mode vie, de prise de conscience de l'image corporelle qui pourrait rendre difficile l'acceptation d'une médication qui peut se révéler fastidieuse, d'autant plus si des effets indésirables apparaissent [4]. La puberté est une période de modifications hormonales surtout sur l'axe hypothalamo-hypophyso-ovariennes. Celles-ci provoquent des changements corporels de la jeune fille. Les conséquences de ces changements hormonaux et métaboliques peuvent avoir une modification de la stabilité de l'épilepsie [5, 6]. La puberté influence sur excitabilité neuronale par modification évolutive de certains syndromes épileptiques idiopathiques en particulier la disparition ou régression de l'épilepsie type absence, épilepsie à pointe centro-temporale (EPCT) et l'apparition d'une épilepsie myoclonique juvénile, l'épilepsie cataméniale. A ce stade, les enfants se comportent comme autonome. La prise en charge doit insister sur l'observance du traitement avant d'évaluer l'efficacité du traitement. L'adolescente et son entourage aient une bonne compréhension de la maladie épileptique. L'éducation, le dialogue, une prise en charge individuelle, un choix judicieux des médicaments s'imposent donc pour favoriser une compliance adéquate et assurer un vécu optimal de l'épilepsie [3, 4].

**Epilepsie et sexualité:** la sexualité constitue un volet important pour toutes les personnes qu'elles soient atteintes ou non d'épilepsies. L'épilepsie affecte aussi sur la sexualité tant sur la difficulté d'avoir un partenaire et sur les troubles sexuels des patients sous antiépileptiques. La maladie épileptique pourrait être source de difficulté pour trouver un partenaire et même la séparation d'un couple. Les troubles sexuels se manifestent soit par angoisse, peur qui entraine la diminution de la fréquence des rapports sexuels et perte de la libido [7]. Ce dernier provoque une réaction du partenaire qui pourrait affecter la vie familiale. A ces effets s'ajoutent l'infertilité du couple qui pourrait interpréter à tord liée à la maladie. Par contre à l'heure actuelle, les effets de l'épilepsie, des crises convulsives et des drogues antiépileptiques sur la fertilité restent toujours en controverses. Il est important d'expliquer au malade, à l'entourage et a la communauté que la plupart des personnes atteintes d'épilepsie peuvent avoir une vie sociale sexuelle et familiale épanouie [7, 8].

**Epilepsie et contraception:** la contraception est un droit absolu de la femme et l'épilepsie n'empêche pas la contraception. Par contre, les médicaments antiépileptiques sont des inducteurs enzymatiques (lamotrigine, valproate de soduim, carbamazépine, epitomax, felbamate). Ils peuvent donc réduire drastiquement l'efficacité de la contraception hormonale chimique [9]. Vous devez surtout de méfier sur les antiépileptiques. La nécessité d'une information éclairée et concertée du gynécologue et neurologue est d'importance capitale pour les méthodes chimiques. Plusieurs méthodes contraceptives sont adaptées aux épileptiques, (Acetate de Medroxyprogesterone injectable, DIU hormonal, DIU au cuivre). En cas de doute, il faut utiliser un préservatif ou un autre moyen de contraceptions mécaniques pour plus de sécurité [10, 11].

**Epilepsie et grossesse:** les femmes présentent des modifications physiologiques qui peuvent altérer sa qualité de vie au cours de la grossesse. Des changements importants qui pouvaient interférer l'augmentation de la fréquence de leurs crises telle qu'une diminution de la concentration plasmatique des antiépileptiques [12, 13]. La diminution de la concentration plasmatique est la conséquence de l'augmentation du volume de distribution de l'organisme maternelle ou la malabsorption digestive du traitement et la mauvaise observance de la femme enceinte qui craint les effets néfastes du traitement sur le fœtus. Une épileptique stable nécessite toujours une surveillance plus approfondie pendant la grossesse. En général au cours de la grossesse, un tiers des patientes rapportent une augmentation de la fréquence des crises. La survenue de crises tonico-cloniques peut entraîner des conséquences graves [13, 14]. La prise en charge des femmes épileptiques enceintes constitue un challenge du fait des modifications pharmacocinétiques et de la tératogénicité des médicaments antiépileptiques. Il impose à gérer l'absence des récidives de la crise convulsive et aussi le développement du fœtus jusqu'à son terme. Avoir une épilepsie équilibrée dans les mois précédant la grossesse est un facteur prédictif de bon équilibre durant la grossesse. Chez les épileptiques, la grossesse doit être préparée par une consultation préconceptionnelle du couple. Le traitement doit être en monothérapie tant que possible et surtout les anciennes molécules. Eviter les molécules connues tératogènes sauf en absence d'autre alternative. Certains médicaments nécessitent une adaptation posologique (lamotrigine, levetiracetam, oxycarbazépine). Une supplémentation acide folique 5mg/j, 2-3mois avant et 4 mois (au moins) après la grossesse est indiquée [12-15].

**Epilepsie et accouchement:** l'accouchement donne lieu à une préparation spécifique. L'équipe médicale doit être bien informée de l'épilepsie surtout si elle n'est pas stabilisée. Tout est mis en œuvre pour éviter le risque de crise pendant l'accouchement. Une épileptique stable ne coure moins de risque de récidive de crise pendant l'accouchement. Il faut faire un accouchement par voie basse si les conditions obstétricales le permettent [13]. L'épilepsie n'augmente pas le risque d'accouchement par césarienne. Cependant pour la prévention du risque hémorragique, la vitamine K1 pendant les 3 semaines avant l'accouchement peut être nécessaires. Le traitement antiépileptique habituel doit être poursuivi pendant le travail et l'accouchement. Le nouveau né recevra une injection de vitamine K1 à dose standard (2mg per os ou IM) ou une dose à risque hémorragique majoré (0,5 à 1mg IM ou IV lente de vitamine K1), si la mère recevait des inducteurs enzymatiques (PHB). L'épilepsie stable ne constitue pas une inquiétude pour l'accouchement [16, 17].

**Epilepsie et allaitement:** le lait maternel est spécialement adapté pour répondre aux besoins du bébé dès sa naissance. Il a donc des bienfaits pour sa santé et son développement. De plus, l'allaitement a aussi des bienfaits pour la mère. Tous les antiépileptiques se retrouvent en une certaine concentration dans le lait maternel. Cependant, ces concentrations restent très faibles [11]. La problématique métabolique demeure pour le nouveau-né. Effectivement, le taux sérique des protéines est faible, son métabolisme hépatique est lent, ce qui peut donc engendrer pendant quelques jours des taux thérapeutiques d’antiépileptiques, d’où la possibilité de quelques effets secondaires tels que: sédation si la mère épileptique est traitée au phénobarbital ou à la primidone et irritabilité si la mère est sous un traitement de benzodiazépines par syndrome de sevrage chez le nouveau-né. Dans la pratique, il n’y a aucune contre-indication pour l’allaitement chez une femme épileptique [16].

## Conclusion

L'épilepsie est une pathologie qui survient à tout âge, n'épargnant aucun sexe. L'influence de la vie sexuelle de l'épilepsie et même l'inverse constitue une préoccupation majeure des praticiens. L'épilepsie et la santé de la reproduction nécessite une bonne connaissance de la maladie épileptique, des antiépileptiques aussi la connaissance des rôles variés et spécifiques surtout des femmes au cours de leur vie: puberté, sexualité, contraception, grossesses et ménopause. Il est important pour les praticiens en neurologie ou en gynécologie obstétrique de ne pas surestimer ou sous-estimer les risques, quelle que soit la situation. La consultation préconceptionnelle, prénatale est de règle car la grossesse doit être préparée chez les épileptiques. Toujours éviter les médicaments clairement tératogènes. Une décision conjointe est nécessaire pour certaines situations difficiles. En respectant ses conditions qu'on pourra mener une vie reproductive heureuse chez nos épileptiques.

## Conflits d’intérêts

Les auteurs ne déclarent aucun conflit d'intérêts.
